# Validity of a New Quantitative Evaluation Method that Uses the Depth of the Surface Imprint as an Indicator for Pitting Edema

**DOI:** 10.1371/journal.pone.0170810

**Published:** 2017-01-27

**Authors:** Haruki Kogo, Jun Murata, Shin Murata, Toshio Higashi

**Affiliations:** 1 Faculty of Rehabilitation Science, Nishikyushu University, Kanzaki-shi, Saga, Japan; 2 Nagasaki University Graduate School of Biomedical Sciences, Nagasaki, Japan; 3 Faculty of Health Science, Kyoto Tachibana University, Yamashina-ku, Kyoto, Japan; Northwestern University Feinberg School of Medicine, UNITED STATES

## Abstract

This study examined the validity of a practical evaluation method for pitting edema by comparing it to other methods, including circumference measurements and ultrasound image measurements. Fifty-one patients (102 legs) from a convalescent ward in Maruyama Hospital were recruited for study 1, and 47 patients (94 legs) from a convalescent ward in Morinaga Hospital were recruited for study 2. The relationship between the depth of the surface imprint and circumferential measurements, as well as the relationship between the depth of the surface imprint and the thickness of the subcutaneous soft tissue on an ultrasonogram, were analyzed using a Spearman correlation coefficient by rank. There was no significant relationship between the surface imprint depth and circumferential measurements. However, there was a significant relationship between the depth of the surface imprint and the thickness of the subcutaneous soft tissue as measured on an ultrasonogram (correlation coefficient 0.736). Our findings suggest that our novel evaluation method for pitting edema, based on a measurement of the surface imprint depth, is both valid and useful.

## Introduction

Generally, edema results when the physiological compensatory functions for excess interstitial fluid are surpassed, resulting in the accumulation of superfluous water [1]. With regard to the pathogeny of edema, several factors may contribute. Increases in vasocapillary hydrostatic pressure and capillary permeability can result in superfluous fluid in the water provision system in the tissue space. Similarly, the water content redistribution system for interstitial fluid may not function sufficiently due to a reduction in plasma osmolarity or a reduction in skin compliance, and in some cases, a mix of these factors may coexist [1]. Edema can be categorized as either pitting edema or non-pitting edema [2]. Almost all cases of edema are pitting edema, and many cases of non-pitting edema are actually lymphedema, in which the tissues proceed to harden. Diseases resulting in symptoms of edema that are encountered at medical institutions vary from internal diseases (e.g., kidney, liver, heart, and endocrine diseases) to orthopedic diseases, central nervous system diseases, and malignant tumors [3]. Edema of the extremities is a somatic symptom that is also commonly observed in daily life.

Although edema is generally identified by patient interview, physical examination, and palpation, it can be difficult to evaluate quantitatively. Palpation is the most convenient method, but it is semi-quantitative and lacks the reproducibility of a measured value. In addition, circumferential measurements [4–7] are often carried out. Applying laterality (bilateral measurements) and variation (repeat measures) is meaningful because one measurement alone cannot be used to quantitatively evaluate edema. Other measurement methods include water bath draining [8–10], ultrasound imaging [11], computed tomography (CT) [12], magnetic resonance imaging (MRI), and lymph scintigraphy [13], among others. However, these evaluation methods result in physical and economic burden to the patient because they require high-priced instruments and are not easy to perform. Recently, the impedance method [14] has been used in the field of obstetrics and gynecology [15]. However, this evaluation method may measure the amount of systemic fluid, and it may vary due to water intake before an examination. For these reasons, conventional evaluation methods are impractical, thus the development of a novel, objective evaluation method that can easily quantify edema is warranted. Furthermore, it is considered to be an essential way of assessing the status of the progress of a disease by quantitatively evaluating the accumulation of interstitial fluid at a specific site.

In the case of pitting edema, if acupressure is performed, a surface imprint remains, unlike the case of swelling or cicatrization. Furthermore, the time required for the surface imprint to recover to its former state is about 1 minute or more for an individual [9]; therefore, a quantitative assessment of the surface imprint will enable physicians to evaluate pitting edema quantitatively. Measuring the depth of the surface imprint is simple and may provide a valuable tool for use in the clinic. We developed a new practical evaluation method for pitting edema and verified the reliability of the method, which uses the depth of a surface imprint as an indicator [3]. Our previous study yielded high intra-rater correlation coefficients (ICC [1,1]: right, 0.91; left, 0.97) and high inter-rater correlation coefficients (ICC [2,1]: right, 0.99; left, 0.97), indicating that the new practical evaluation is reliable. Therefore, this practical evaluation method can measure a surface imprint easily, and it may be a useful instrument in the clinic. Thus, the purpose of the present study was to examine the validity of this evaluation method by comparing it to the circumference measurement method, which was chosen because it is often used in clinical practice, and an ultrasound imaging measurement method, which was chosen for ethical reasons since it is comparatively easy to obtain approval from subjects for this non-aggressive and non-invasive measure.

## Subjects and Methods

### Study 1: Relationship of the surface imprint depth measurement method to the circumference measurement method

Fifty-one patients (102 legs; 32 men, 19 women; average age, 69.8 ± 13.9 years) from a convalescent ward in Maruyama Hospital (Table 1) were recruited. All subjects were diagnosed as having edema by physical examination and palpation, and their general condition was comparatively stable. Patients with dementia and foot vulnerabilities, and those who were unavailable during the study period were excluded.

**Table 1:** Subjects’ characteristics (n = 51).

Age, years	69.8 ± 13.9
Sex, (male/female)	32/19
Height, cm	159.6 ± 8.5
Weight, kg	58.2 ± 12.4
BMI, kg/m^2^	22.7 ± 3.6
Heart disease, n	20
Central nervous disease, n	51
Pancreopathy, n	4
Orthopedic issues, n	3
Kidney disease, n	4
Pulmonary disease, n	0

BMI, body mass index. Data for age, height, weight, and BMI are presented as the mean ± standard deviation.

The study was performed in accordance with the Declaration of Helsinki. Details of the study were sufficiently explained to the subjects, and all participants signed informed consent forms. The study was also approved by the Ethics Committee of the University of Nishikyushu (approval no.: H25-6) and Maruyama Hospital.

#### Measurement of the depth of the surface imprint

**Fig 1:**
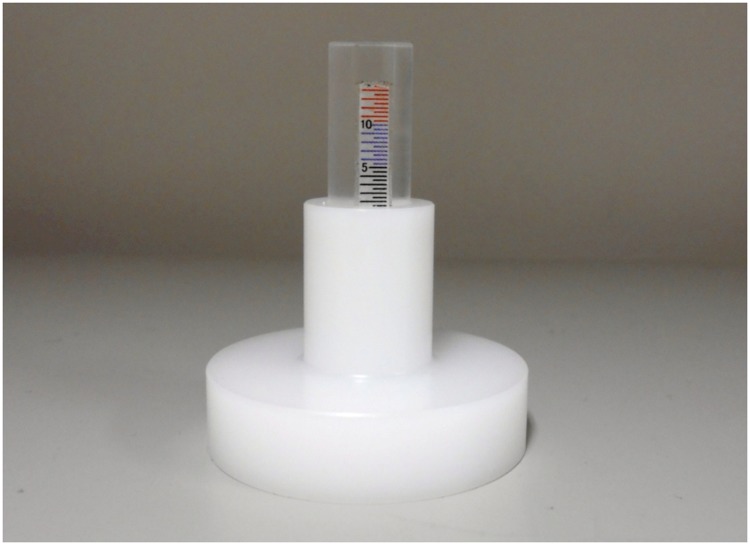
Measurement instrument.

**Fig 2:**
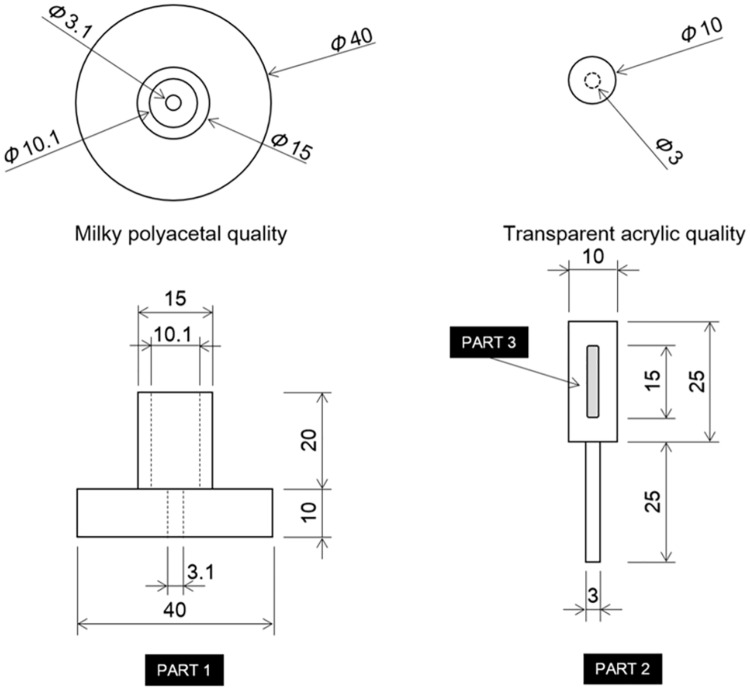
Specifications of the measurement instrument.

**Fig 3:**
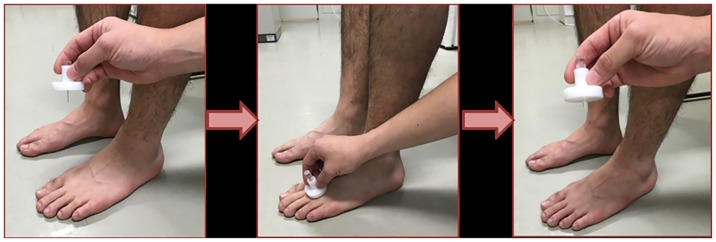
Visualization process of the instrument for the surface imprint.

Subjects sat on the edge of a chair and were asked to lightly press the soles of their feet into the floor. Assessments were performed for the right and left sides in a relaxed state so that muscular contraction was not initiated. All measurements were performed in the morning, just prior to examination or medical treatment. The surface imprint was made with a digital force gauge (FG-5005, Mother Tool Co., Ltd., Nagano, Japan), which was equipped with a 25-mm diameter globe attachment made of rubber, by pressing down with approximately 20 N of compressive force. The examiner pressed down with a digital force gauge for 10 seconds on the top of each foot in the central region, along the line that connects the first metatarsal head and the fifth metatarsal head, and recorded the measurement after 10 seconds. The measurement instrument (edema gauge, KM-212-003, Unique Medical Company, Tokyo, Japan) used to measure the depth of the surface imprint has been previously described [3] (Fig 1). Specifications of the measurement instrument are shown in Fig 2, and the visualization process of the instrument for the surface imprint is shown in Fig 3. Part 1 is held with the thumb, third finger, and fourth finger. Then, using the forefinger, part 2 is depressed to the lowest part. After the tip of part 2 gently contacts the deepest region of the surface imprint, part 1 is pushed down until contact is made with the portion surrounding the surface imprint. After part 1 has been positioned, the measurement instrument separates from the measurement part for visualization. The examiner was careful during the measurement to avoid depressing the tip of part 2 too deeply into the surface imprint. Thereafter, the examiner measured the depth of the surface imprint.

The examiner had 10 years of clinical experience, with high intra-rater correlation coefficients (ICC [1,1]: right, 0.91; left, 0.97) in a previous work [3]. The examiner collected all measurements for a given subject. Each measurement was performed twice, and the values were averaged.

#### Circumferential measurements

Subjects assumed a long sitting position on the treatment table, with the feet extended beyond the table at the level of the midcalf, and they remained in this position. Landmarks were marked on the first metatarsal head and the fifth metatarsal head. Circumferential measurements were taken using a tape measure that was 8-mm wide and 1.5-m long. The circumferential measurements were taken around the foot along the line that connects the first metatarsal head and the fifth metatarsal head, according to the method described in a prior study [7]. The examiner was the same person who measured the depth of the surface imprint and collected all measurements for a given subject.

### Study 2: Relationship between the surface imprint depth measurement method and ultrasound imaging

Forty-seven patients (94 legs; 21 men, 26 women; average age, 81.9 ± 13.3 years) from a convalescent ward in Morinaga Hospital (Table 2) were recruited. All subjects were diagnosed as having edema by physical examination and palpation, and their general condition was comparatively stable. Patients with dementia and foot vulnerabilities, and those who were unavailable during the study period were excluded.

**Table 2:** Subjects’ characteristics (n = 47).

Age, years	80.9 ± 10.9
Sex, (male/female)	21/26
Height, cm	154.0 ± 10.9
Weight, kg	55.4 ± 16.4
BMI, kg/m^2^	23.2 ± 5.7
Heart disease, n	15
Central nerve disease, n	24
Pancreopathy, n	11
Orthopedic issues, n	30
Kidney disease, n	5
Pulmonary disease, n	2

BMI, body mass index. Data for age, height, weight, and BMI are presented as the mean ± standard deviation.

The study was performed in accordance with the Declaration of Helsinki. Details of the study were sufficiently explained to the subjects, and all participants signed informed consent forms. The study was approved by the Ethics Committee of the University of Nishikyushu (approval no.: H25-6) and Morinaga Hospital.

#### Measurement of the depth of the surface imprint

The procedure used in study 2 was identical to that used in study 1. The examiner had 10 years of clinical experience and abundant experience in the examination of ultrasound imaging. The same examiner collected all measures for a given subject.

#### Measurement of the thickness of the subcutaneous tissue by ultrasound imaging

**Fig 4:**
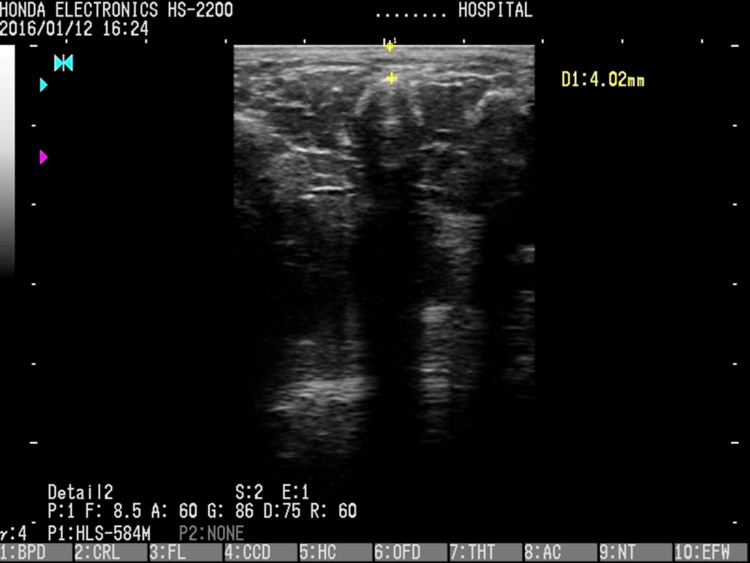
Measure of the thickness of the subcutaneous soft tissue by ultrasound imaging.

The same measurement region used for the surface imprint depth measurement was selected. An ultrasound imaging apparatus (HS-2200, Honda Electric, Inc., Toyohashi, Japan) with a linear probe (HLS-575M, Honda Electric, Inc.) was used, and measurements were performed at a 7.5-MHz frequency in B-mode. The thickness of the subcutaneous soft tissue was measured from the upper part of the hypodermic fat layer to the upper part of the third metatarsal head on the top of the feet (Fig 4). Since ultrasonography evaluation may be influenced by the examiner’s technique, the same examiner performed all measurements. This examiner was the same person who measured the depth of the surface imprint, and collected all measures for a given subject. Each measurement was performed twice, and the values were averaged.

Landmark: From the upper part of the hypodermic fat layer to the upper part of the third metatarsal head

### Statistical analysis

After analyzing the bivariate normality of each measure using the Kolmogorov-Smirnov test, the relationship between the depth of the surface imprint and the circumference was analyzed, as well as the relationship between the depth of the surface imprint and the thickness of the subcutaneous soft tissue, using the Spearman correlation coefficient by rank. For statistical analysis, the level of significance was set at 5%, and SPSS, version 20 for Windows (IBM Corp., Armonk, NY, USA) was used for analysis. In addition, to compute the 95% confidence interval for the coefficient between the depth of the surface imprint and the thickness of the subcutaneous soft tissue, VassarStats was used [16].

## Results and Discussion

### Results

Characteristics of the study subjects are shown in Tables 1 and 2. For study 1, with regard to the relationship between the depth of the surface imprint and the circumference, the average surface imprint depth was 2.9 ± 1.1 mm and the average circumference was 237.1 ± 13.8 mm. There was no significant relationship between the surface imprint depth and circumference (Table 3).

**Table 3:** Correlation coefficients between the depth of the surface imprint, circumference, and thickness of the subcutaneous tissue.

	Circumference	Thickness of the subcutaneous soft tissue
Depth of the surface imprint	-0.025	0.736**

The 95% confidence interval for the coefficient between the depth of the surface imprint depth and the thickness of the subcutaneous soft tissue is 0.627 to 0.816.

** p < 0.01

**Fig 5:**
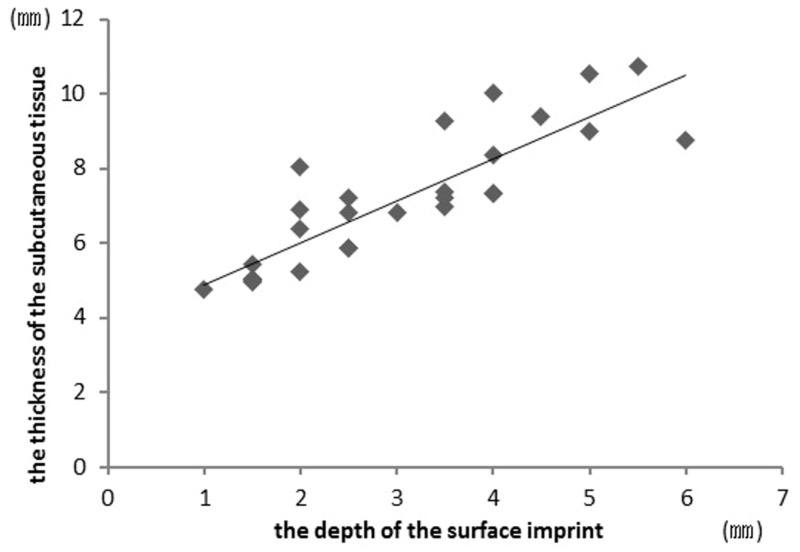
Correlation diagram of the depth of the surface imprint and the thickness of the subcutaneous tissue.

For study 2, with regard to the relationship between the depth of the surface imprint and the thickness of the subcutaneous soft tissue by ultrasound imaging, the average surface imprint depth was 2.8 ± 1.4 mm and the average thickness of the subcutaneous soft tissue was 7.5 ± 1.4 mm. The correlation coefficient was 0.736, and a significant relationship between the depth of the surface imprint and the thickness of the subcutaneous soft tissue was demonstrated (Table 3) (Fig 5).

## Discussion

While examining the validity of the surface imprint depth as a practical evaluation method for pitting edema, we found that there was no significant relationship between surface imprint depth and circumferential measurements. In a previous study, Taylor et al. verified the reliability and validity of circumferential measurements by comparing upper extremity circumferential measurements using a tape measure to upper extremity volumetric measurements using water displacement in subjects with lymphedema [4]. However, these measurement methods are used to assess the gross tissues, including bone, and soft tissues, such as muscle and fat. Moreover, these measurement methods are influenced by the physical constitution of the individual being tested, and basic measured values cannot be compared directly between individuals because a single measurement cannot be used to determine the existence of pitting edema. Since a significant relationship was not verified between the surface imprint depth and circumference in this study, surface imprint depth measurements may be a reflection of a different evaluative method, which provides different information than circumferential measurements.

Moreover, there was a significant relationship between the depth of the surface imprint and the thickness of the subcutaneous soft tissue. According to a previous study evaluating lymphedema in patients with malignant tumors, the thickness of the subcutaneous soft tissue measured by MRI or ultrasound imaging is considered to be a parameter of quantitative evaluation [13, 17], and ultrasound imaging is considered to be a common objective evaluation method [18]. Consequently, the ultrasound imaging method is considered a valid evaluation method, and its use has been prevalent in recent years. In a previous study comparing patients with lymphedema and healthy subjects, Dimakakos et al. measured the thickness of the subcutaneous soft tissue using ultrasound imaging [19] and reported having observed a significant increase in the thickness of the subcutaneous fat, although there was no significant difference in skin thickness between the patients with lymphedema and the healthy controls. In the same study, measurement of the thickness of subcutaneous soft tissue by MRI also showed that patients with lymphedema exhibit a significant increase in subcutaneous soft tissue compared to healthy subjects. Furthermore, in another study on patients with lymphedema, Niimi et al. evaluated structural changes of the subcutaneous soft tissue by the ultrasound imaging method [11] and proved that the volume of subcutaneous soft tissue correlated with fluid accumulation. In consideration of these previous studies and the relationship between the surface imprint depth and subcutaneous soft tissue thickness by ultrasound imaging, we believe that surface imprint depth measurements are a valid quantitative evaluation method for pitting edema.

## Conclusions

As our findings suggest that a significant relationship between the depth of the surface imprint and the thickness of the subcutaneous soft tissue is present, this new evaluation method could be useful (Table 3 and Fig 5). However, our findings provide evidence for the validity of a surface imprint depth measurement, which is a limitation of the interpretation of the results of this study. Therefore, future research needs to be performed to confirm this relationship and further explore the validity of the measure. It is a limitation of this study that we cannot actually measure the amount of interstitial fluid in the subcutaneous soft tissue of the specified region. As surface imprint depth measurements have been shown to reflect the quantity of interstitial fluid, future research may focus on how surface imprint depth may be used to actually measure the amount of interstitial fluid in a specified region. Unlike other evaluation methods, the ultrasound imaging and circumference measurement methods depend on the examiner’s skill. Therefore, in this study, we used a single examiner with experience. However, future studies may need to evaluate how the method functions in a general population of clinicians by sampling clinicians to measure the same set of patients.
